# Chitosan-Mediated Expression of *Caenorhabditis elegans fat-1* and *fat-2* in *Sparus aurata*: Short-Term Effects on the Hepatic Fatty Acid Profile, Intermediary Metabolism, and Proinflammatory Factors

**DOI:** 10.3390/md23110434

**Published:** 2025-11-13

**Authors:** Yuanbing Wu, Ania Rashidpour, Wenwen Duan, Anna Fàbregas, María Pilar Almajano, Isidoro Metón

**Affiliations:** 1 Secció de Bioquímica i Biologia Molecular, Departament de Bioquímica i Fisiologia, Facultat de Farmàcia i Ciències de l’Alimentació, Universitat de Barcelona, Joan XXIII 27-31, 08028 Barcelona, Spain; wuuanbing@gmail.com (Y.W.); aniyarashidpoor2017@gmail.com (A.R.); wenwenduan@ub.edu (W.D.); 2 Departament de Farmàcia i Tecnologia Farmacèutica, i Fisicoquímica, Facultat de Farmàcia i Ciències de l’Alimentació, Universitat de Barcelona, Joan XXIII 27-31, 08028 Barcelona, Spain; anna5791@gmail.com; 3 Departament d’Enginyeria Química, Universitat Politècnica de Catalunya, Diagonal 647, 08028 Barcelona, Spain; m.pilar.almajano@upc.edu

**Keywords:** chitosan, inflammation, intermediary metabolism, omega-3 long-chain polyunsaturated fatty acids, *Sparus aurata*

## Abstract

A single dose of chitosan-tripolyphosphate (TPP) nanoparticles carrying expression plasmids for fish codon-optimized *Caenorhabditis elegans fat-1* and *fat-2* was intraperitoneally administered to gilthead seabream (*Sparus aurata*) to stimulate the biosynthesis of omega-3 long-chain polyunsaturated fatty acids (*n*-3 LC-PUFA) and evaluate subsequent short-term effects on liver intermediary metabolism and immunity. Seventy-two hours post-injection, the upregulation of *fat-1* elevated eicosapentaenoic acid (EPA), docosahexaenoic acid (DHA), and total *n*-3 fatty acids in the liver, while *fat-2* enhanced DHA and *n*-3 fatty acids. Co-expression of *fat-1* and *fat-2* increased EPA, DHA, PUFA, and the total *n*-6 and *n*-3 LC-PUFA, while reducing plasma triglycerides. The expression of *fat-1* and *fat-2* suppressed hepatic lipogenesis by downregulating *srebf1* and *pparg*, and consequently key genes in fatty acid synthesis (*acaca*, *acacb*, *fasn*, *scd1*, and *fads2*). In contrast, the co-expression of *fat-1* and *fat-2* upregulated *hnf4a*, *chrebp*, and *pfkl*, a rate-limiting enzyme in glycolysis. Furthermore, *fat-1* and *fat-2* reduced hepatic proinflammatory markers such as *tnfa* and *nfkb1*. In addition to enhancing EPA and DHA biosynthesis, promoting glycolysis, and suppressing lipogenesis, our findings suggest that the short-term expression of *C. elegans fat-1* and *fat-2* in the liver may also reduce inflammation and, therefore, could impact the health and growth performance of cultured fish.

## 1. Introduction

In recent years, the aquaculture industry has made intensive efforts to replace fish oil in aquafeeds, at least partially, with other fat sources, such as vegetable oils. However, plants contain higher levels of omega-6 fatty acids than omega-3 fatty acids and are devoid of omega-3 long-chain polyunsaturated fatty acids (*n*-3 LC-PUFA) such as eicosapentaenoic acid (EPA) and docosahexaenoic acid (DHA). As a result, the replacement of fish oil with vegetable oils significantly reduces *n*-3 LC-PUFA in fish flesh [1]. Although dietary *n*-3 LC-PUFA is required for optimal growth, marine fish, especially carnivorous fish, exhibit low ability to synthesize *n*-3 LC-PUFA [2]. In general, vertebrates are devoid of the Δ12/*n*-6 and Δ15/*n*-3 desaturases required to synthesize linoleic acid (LA) from oleic acid (OA) and α-linolenic acid (ALA) from LA. Therefore, vertebrates, including bony fish, can only synthesize a small portion of LC-PUFA at insufficient rates to meet physiological demands [3,4]. In addition to storing energy, LC-PUFA and their derivatives have pivotal roles in growth and development, serving as biologically active moieties in membrane phospholipids, being substrates for signalling molecules, and regulating gene transcription [5]. The mechanisms by which *n*-3 LC-PUFA regulate energy metabolism and immunity in fish, two processes closely linked to growth, have yet to be fully elucidated.

A competitive relationship has been found between the synthesis of *n*-3 and *n*-6 PUFA series due to shared desaturases and elongases in their respective biosynthetic pathways [6]. Given that oxidized derivatives of the *n*-6 LC-PUFA precursor, LA, are proinflammatory factors [7], and *n*-3 LC-PUFA are valued for their anti-inflammatory effects, the impact of the physiological ratio between *n*-3 and *n*-6 PUFA on inflammation is receiving increasing attention. Compared to vegetable oils, fish oil has a lower *n*-6 to *n*-3 PUFA ratio. Therefore, dietary replacement of fish oil with vegetable oils in marine fish was shown to upregulate the expression of proinflammatory genes such as myeloid differentiation primary response 88 (*myd88*), tumor necrosis factor a (*tnfa*), interleukin 1b (*il1b*), and the nuclear factor kappa-light-chain-enhancer of activated B cells (*nfkb*) [8,9].

Although absent in fish, Δ15/*n*-3 and Δ12/*n*-6 desaturases have been identified in some invertebrates such as the roundworm *Caenorhabditis elegans*, where they are referred to as *fat-1* and *fat-2*, respectively. To alleviate the fish oil dependency of aquafeeds, the efficient conversion of *n*-6 PUFA to *n*-3 PUFA has been reported in transgenic models, such as zebrafish [10] and common carp [11], expressing codon-optimized *C. elegans fat-1* and/or *fat-2*. The *n*-3 LC-PUFA content and *n*-6 PUFA to *n*-3 PUFA conversion were further intensified in zebrafish and pigs through the double transgenesis of *fat-1* and *fat-2* [1,12]. Nevertheless, the effects of *C. elegans fat-1* and *fat-2* on intermediary metabolism and inflammation in transgenic animals remain scarce.

Chitosan is a biomaterial derived from chitin, a natural polysaccharide that is a primary component of invertebrate exoskeletons and is abundantly found in nature. Low toxicity, biocompatibility, and biodegradability make chitosan a highly suitable carrier for delivering nucleic acids. Using sodium tripolyphosphate (TPP), ionic gelation of chitosan-TPP-DNA has garnered growing attention due to its mild processing conditions, nanoscale production capabilities, and cost-effectiveness in gene delivery for biotechnological applications in fish [13]. Gilthead seabream (*Sparus aurata*) is a carnivorous fish that accounted for 38.3% of total European aquaculture production of marine fish in 2022 (https://www.fao.org/fishery/en/fishstat, accessed on 9 January 2025). We recently demonstrated that regular supplementation with chitosan-TPP-DNA nanoparticles resulted in the long-term sustained hepatic expression of fish codon-optimized *C. elegans fat-1* and *fat-2*, leading to enhanced *n*-3 LC-PUFA synthesis and improved growth performance in *S. aurata* [14]. To advance the current understanding of the temporal molecular events triggered by *C. elegans fat-1* and *fat-2* in the fish liver, the present study aimed to investigate the short-term effects of chitosan-mediated hepatic expression of fish codon-optimized *C. elegans fat-1* and *fat-2* on intermediary metabolism and inflammation in *S. aurata.*

## 2. Results

### 2.1. Effect of Intraperitoneal Administration of Chitosan-TPP Nanoparticles Encapsulating pSG5-FAT-1 and pSG5-FAT-2 on fat-1 and fat-2 mRNA Levels in S. aurata Liver

**Figure 1:**
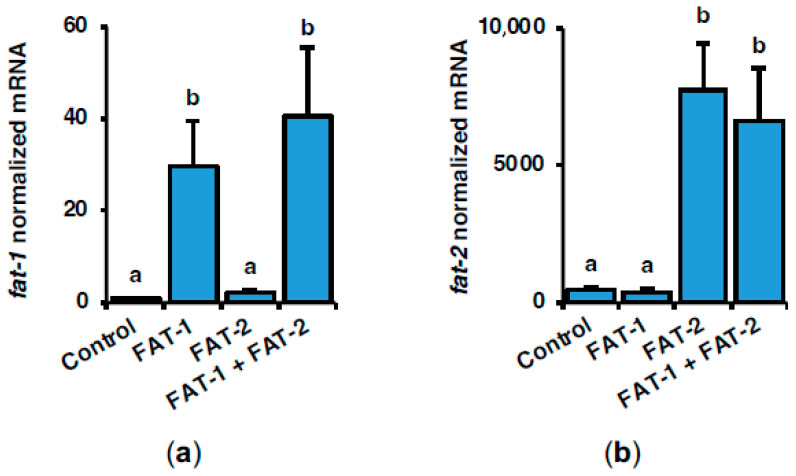
Short-term effects of intraperitoneal administration of chitosan-TPP nanoparticles encapsulating pSG5 (control), pSG5-FAT-1, pSG5-FAT-2, and pSG5-FAT-1 + pSG5-FAT-2 on the mRNA levels of fish-codon optimized *C. elegans fat-1* (**a**) and *fat-2* (**b**) in *S. aurata* liver. Seventy-two hours post-treatment, fish were sacrificed, and the liver was collected. Following RNA isolation from liver samples, exogenous *fat-1* and *fat-2* expression was assayed by RT-qPCR, normalized to the geometric mean of *S. aurata 18s*, *actb*, and *eef1a* mRNA levels, and expressed as mean ± SEM (*n* = 5). Different letters indicate distinct homogeneous subsets determined by the post hoc test (*p* < 0.05).

Chitosan-TPP was complexed with pSG5 (control), pSG5-FAT-1, pSG5-FAT-2, and pSG5-FAT-1 + pSG5-FAT-2 by ionic gelation [14]. No significant differences in particle size were found between naked chitosan-TPP (215 nm ± 20) and chitosan-TPP-DNA (263 nm ± 74), expressed as mean ± SEM (*n* = 3). DNA incorporation significantly decreased *Z* potential from 38 mV ± 1 to 12 mV ± 1, expressed as mean ± SEM (*n* = 3). Seventy-two hours after intraperitoneal administration of a single dose of chitosan-TPP nanoparticles encapsulating either pSG5-FAT-1 or pSG5-FAT-2, the mRNA levels of fish codon-optimized *fat-1* and *fat-2* were assayed by RT-qPCR in the liver of *S. aurata*. Compared with control fish (treated with empty plasmid, pSG5), the hepatic mRNA abundance of *fat-1* in fish administered with pSG5-FAT-1 and pSG5-FAT-1 + pSG5-FAT-2 was 34.1- and 46.7-fold significantly higher, respectively, while *fat-2* mRNA levels in fish treated with pSG5-FAT-2 and pSG5-FAT-1 + pSG5-FAT-2 increased 17.7- and 15.1-fold (Figure 1a,b).

### 2.2. Effect of Chitosan-Mediated Expression of fat-1 and fat-2 on S. aurata Serum Metabolites and Hepatic Fatty Acid Profile

**Figure 2:**
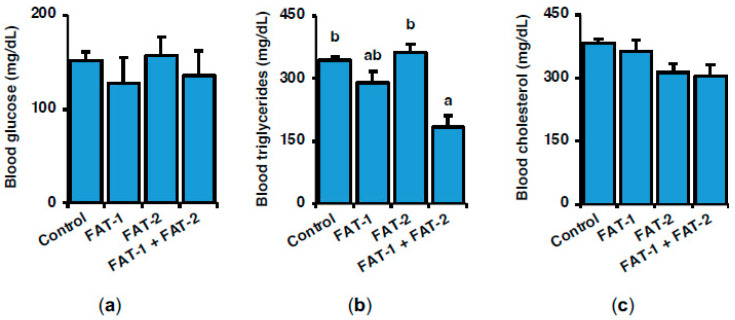
Short-term effects of intraperitoneal administration of chitosan-TPP nanoparticles encapsulating pSG5 (control), pSG5-FAT-1, pSG5-FAT-2, and pSG5-FAT-1 + pSG5-FAT-2 on serum (**a**) glucose, (**b**) triglycerides, and (**c**) cholesterol in *S. aurata*. Seventy-two hours post-treatment, fish were sacrificed, and blood was collected. Values are represented as mean ± SEM (*n* = 4–5). Different letters indicate distinct homogeneous subsets determined by the post hoc test (*p* < 0.05).

Figure 2 shows the effect of hepatic expression of fish codon-optimized *fat-1* and *fat-2* on serum metabolites, including glucose, triglycerides, and cholesterol, 72 h after intraperitoneal administration of a single dose of chitosan-TPP-DNA nanoparticles. Blood glucose and cholesterol were not significantly affected by any of the treatments (Figure 2a–c), while co-expression of *fat-1* and *fat-2* significantly decreased triglycerides to 53.4% of control levels (Figure 2b).

**Figure 3:**
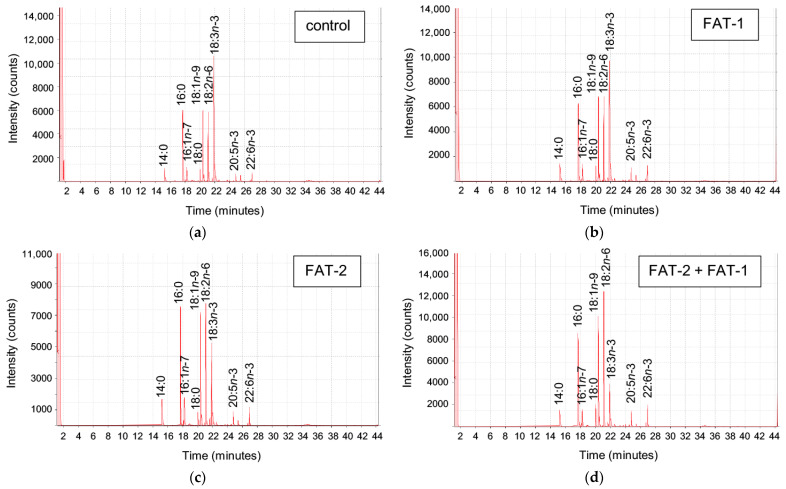
Fatty acid methyl ester analysis of liver samples from *S. aurata*. Representative chromatograms obtained by gas chromatography performed on fish treated with chitosan-TPP nanoparticles complexed with (**a**) pSG5 (control), (**b**) pSG5-FAT-1, (**c**) pSG5-FAT-2, and (**d**) pSG5-FAT-1 + pSG5-FAT-2. Fatty acids with a composition higher than 1% are indicated.

The effect of chitosan-TPP nanoparticles carrying pSG5 (control), pSG5-FAT-1, pSG5-FAT-2, and pSG5-FAT-1 + pSG5-FAT-2 on the fatty acid profile of *S. aurata* liver was analyzed 72 h post-administration (Figure 3).

Among fatty acids identified (30) and those representing higher than 1%, myristic acid (14:0), palmitoleic acid (16:1*n*-7), LA (18:2*n*-6c), EPA (20:5*n*-3), DHA (22:6*n*-3), PUFA, and total *n*-6 and *n*-3 fatty acids were significantly affected by the treatments. Specifically, *fat-1* expression increased palmitoleic acid 1.6-fold and EPA, DHA, and total *n*-3 fatty acids 1.5-fold, while *fat-2* increased palmitoleic acid (2.0-fold), DHA (1.6-fold), and total *n*-3 fatty acids (1.5-fold). Compared to the control fish, co-expression of *fat-1* and *fat-2* increased palmitoleic acid (1.8-fold), LA (1.5-fold), EPA (1.6-fold), DHA (2.0-fold), PUFA (1.5-fold), total *n*-6 fatty acids (1.5-fold), and total *n*-3 fatty acids (1.7-fold) (Table 1).

### 2.3. Effect of Chitosan-Mediated Expression of fat-1 and fat-2 on Key Genes Related to Lipid and Glucose Metabolism

**Figure 4:**
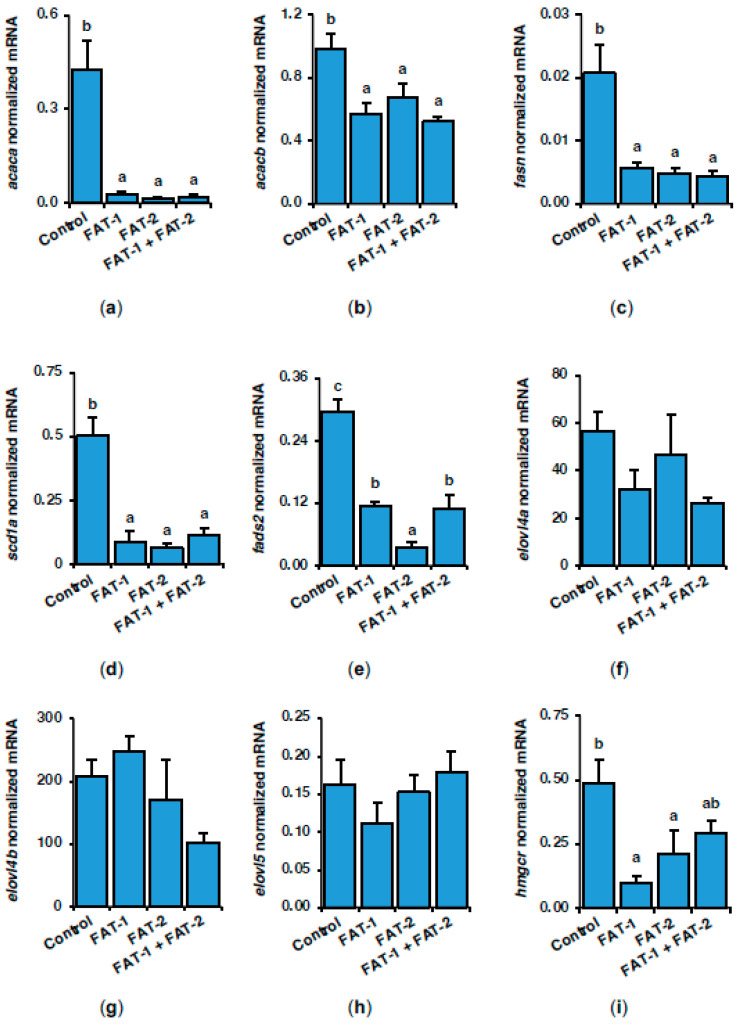
Short-term effects of intraperitoneal administration of chitosan-TPP nanoparticles encapsulating pSG5 (control), pSG5-FAT-1, pSG5-FAT-2, and pSG5-FAT-1 + pSG5-FAT-2 on the expression of key enzymes in *de novo* lipogenesis in the liver of *S. aurata*. Seventy-two hours post-treatment, fish were sacrificed, and the liver was collected. (**a**–**i**) Following RNA isolation from liver samples, gene expression was assayed by RT-qPCR, normalized to the geometric mean of *S. aurata 18s*, *actb*, and *eef1a* mRNA levels, and expressed as mean ± SEM (*n* = 5). Different letters indicate distinct homogeneous subsets determined by the post hoc test (*p* < 0.05).

To shed light on the short-term effects on lipogenic genes resulting from the hepatic expression of fish codon-optimized *fat-1* and *fat-2*, the expression of key enzymes in fatty acid and LC-PUFA synthesis was assayed by RT-qPCR in *S. aurata* liver. Compared to controls, treatment with *fat-1*, *fat-2* and *fat-1* + *fat-2* significantly downregulated lipogenic genes, including acetyl-CoA carboxylase 1 (*acaca*; to 6.8%, 3.4%, and 4.9% of control values, respectively), acetyl-CoA carboxylase 2 (*acacb*; to 57.7%, 68.7%, and 53.0%), fatty acid synthase (*fasn*; to 27.4%, 23.3%, and 21.5%), stearoyl-CoA desaturase-1a (*scd1a*; to 17.8%, 12.7%, and 22.9%), and acyl-CoA 6-desaturase (*fads2*; to 38.9%, 11.9%, and 36.8%) (Figure 4a–e). A similar non-significant trend was found for elongation of very long chain fatty acids protein 4a (*elovl4a*) and elongation of very long chain fatty acids protein 4b (*elovl4b*) (Figure 4f,g). No significant effects were observed in elongation of very long chain fatty acids protein 5 (*elovl5*) mRNA levels (Figure 4h). Although not statistically significant for *fat-1* + *fat-2*, a trend toward decreased 3-hydroxy-3-methylglutaryl-coenzyme A reductase (*hmgcr*) expression to 20.1%, 43.4%, and 59.7% of the control values, respectively, was observed (Figure 4i).

**Figure 5:**
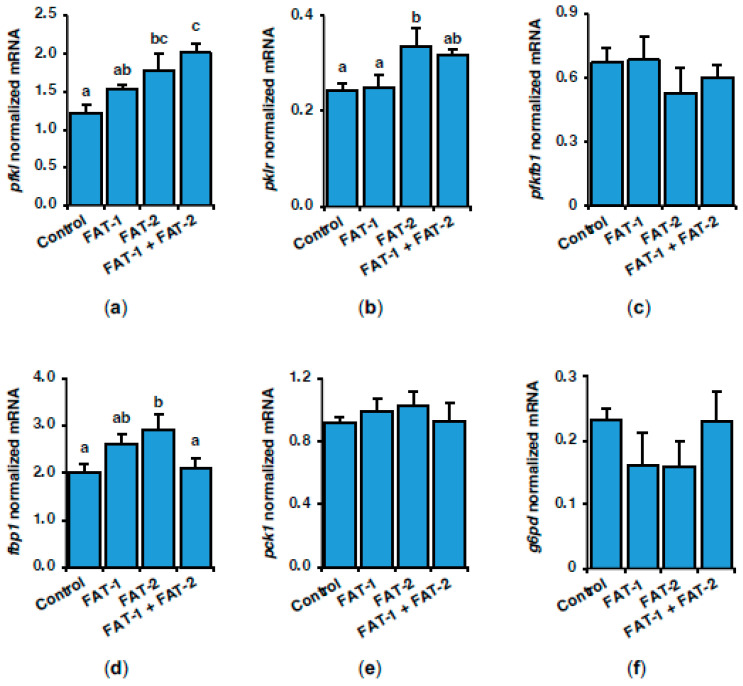
Short-term effects of intraperitoneal administration of chitosan-TPP nanoparticles encapsulating pSG5 (control), pSG5-FAT-1, pSG5-FAT-2, and pSG5-FAT-1 + pSG5-FAT-2 on the expression of key enzymes in glycolysis-gluconeogenesis and the PPP in the liver of *S. aurata*. Seventy-two hours post-treatment, fish were sacrificed, and the liver was collected. (**a**–**f**) Following RNA isolation from liver samples, gene expression was assayed by RT-qPCR and normalized to the geometric mean of *S. aurata 18s*, *actb*, and *eef1a* mRNA levels. Expression levels and enzyme activity are presented as mean ± SEM (*n* = 5). Different letters indicate distinct homogeneous subsets determined by the post hoc test (*p* < 0.05).

Given that hepatic lipogenesis is closely related to glucose metabolism, we also analyzed the expression of genes encoding key enzymes in glycolysis–gluconeogenesis and the pentose phosphate pathway (PPP) in response to the hepatic expression of *fat-1* and *fat-2* in *S. aurata*. Figure 5 shows the effect of *fat-1* and *fat-2* expression on liver mRNA levels of rate-limiting enzymes in glycolysis-gluconeogenesis and PPP, 72 h following nanoparticle administration. Compared to control values, the hepatic expression of *fat-2* upregulated 6-phosphofructo-1-kinase (*pfkl*; 1.5-fold), pyruvate kinase (*pklr*; 1.4-fold), and fructose-1,6-bisphosphatase (*fbp1*; 1.5-fold). Co-expression of *fat-1* and *fat-2* also induced the mRNA abundance of *pfkl* (1.7-fold) (Figure 5a,b,d). No significant effects were observed in 6-phosphofructo-2-kinase/fructose-2,6-bisphosphatase (*pfkfb1*), phosphoenolpyruvate carboxykinase (*pck1*), and glucose-6-phosphate dehydrogenase (*g6pd*) expression (Figure 5c,e,f).

**Figure 6:**
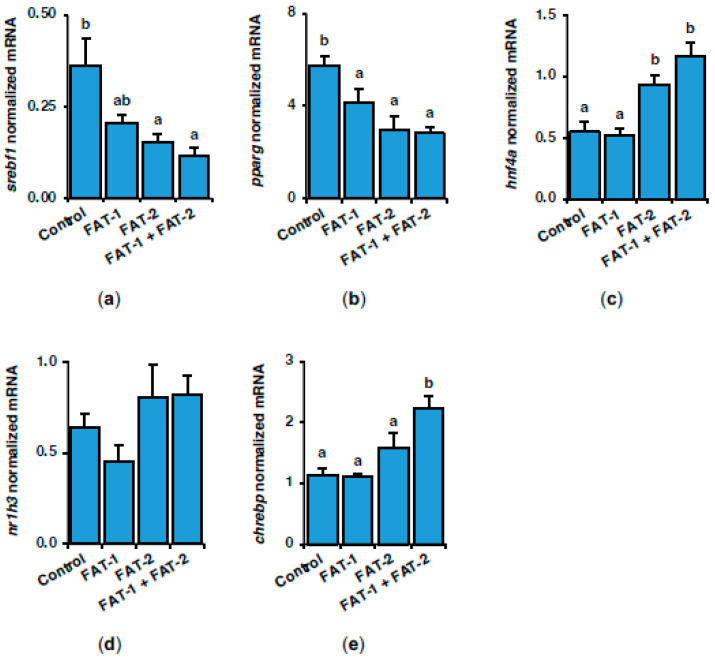
Short-term effects of intraperitoneal administration of chitosan-TPP nanoparticles encapsulating pSG5 (control), pSG5-FAT-1, pSG5-FAT-2, and pSG5-FAT-1 + pSG5-FAT-2 on the expression of key transcription factors in lipogenesis and carbohydrate metabolism in the liver of *S. aurata*. Seventy-two hours post-treatment, fish were sacrificed, and the liver was collected. (**a**–**e**) Following RNA isolation from liver samples, gene expression was assayed by RT-qPCR, normalized to the geometric mean of *S. aurata 18s*, *actb*, and *eef1a* mRNA levels, and expressed as mean ± SEM (*n* = 5). Different letters indicate distinct homogeneous subsets determined by the post hoc test (*p* < 0.05).

To further address the short-term effects resulting from expressing fish codon-optimized *fat-1* and *fat-2* in the liver of *S. aurata*, the mRNA levels of key transcription factors involved in controlling the expression of genes related to lipogenesis and glucose metabolism were also evaluated. Expression of *fat-1*, *fat-2* and *fat-1* and *fat-2* significantly downregulated peroxisome proliferator-activated receptor gamma (*pparg*) to 72.3%, 52.0% and 49.0% of control values, respectively, while treatment with *fat-2* and *fat-1* + *fat-2* also downregulated hepatic sterol regulatory element-binding protein 1 (*srebf1*) to 42.1% and 32.3% of control values (Figure 6a,b). Treatment with *fat-2* and *fat-1* + *fat-2* increased the mRNA levels of hepatocyte nuclear factor 4 alpha (*hnf4a*) (1.7-fold and 2.1-fold of the control values, respectively) and caused similar non-significant effects on oxysterols receptor LXR-alpha (*nr1h3*) expression (Figure 6c,d). Co-expression of *fat-1* and *fat-2* upregulated 1.9-fold the expression levels of carbohydrate-responsive element-binding protein (*chrebp*) (Figure 6e).

### 2.4. Effect of Chitosan-Mediated Expression of fat-1 and fat-2 on Genes Related to Inflammation

**Figure 7:**
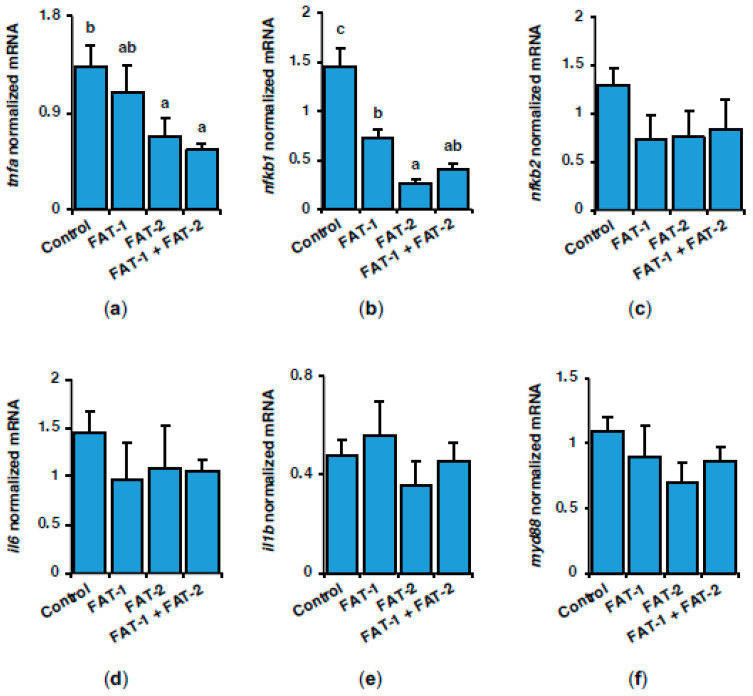
Short-term effects of intraperitoneal administration of chitosan-TPP nanoparticles encapsulating pSG5 (control), pSG5-FAT-1, pSG5-FAT-2, and pSG5-FAT-1 + pSG5-FAT-2 on the expression of pro-inflammatory factors in the liver of *S. aurata*. Seventy-two hours post-treatment, fish were sacrificed, and the liver was collected. (**a**–**f**) Following RNA isolation from liver samples, gene expression was assayed by RT-qPCR, normalized to the geometric mean of *S. aurata 18s*, *actb*, and *eef1a* mRNA levels, and expressed as mean ± SEM (*n* = 5). Different letters indicate distinct homogeneous subsets determined by the post hoc test (*p* < 0.05).

Previous studies demonstrated that *n*-3 LC-PUFA inhibits the activation of immune cells. Hence, in the present study, the expression of genes related to inflammation was also addressed in the liver of *S. aurata*. Seventy-two hours following treatment with chitosan-TPP-DNA nanoparticles, a significant decrease in the mRNA levels of nuclear factor NF-kappa-B p105 subunit (*nfkb1*) was found in the liver of fish administered with *fat-1*, *fat-2*, or *fat-1* + *fat-2* (to 49.9%, 17.8%, and 27.7% of control values, respectively), while the expression of tumor necrosis factor (*tnfa*) also decreased after treatment with *fat-2* and *fat-1* + *fat-2* nanoparticles (to 51.1% and 41.4% of control values, respectively) (Figure 7a,b). Although not significant, a similar trend was observed in regard to the expression of nuclear factor NF-kappa-B p100 subunit (*nfkb2*)*,* interleukin-6 (*il6*), and myeloid differentiation primary response protein MyD88 (*myd88*), while no significant differences were noticed for interleukin-1 beta (*il1b*) (Figure 7c–f).

## 3. Discussion

To better understand the molecular events triggered by hepatic expression of *C. elegans fat-1* and *fat-2* in *S. aurata*, the effects of a single intraperitoneal administration of chitosan-TPP nanoparticles loaded with expression plasmids carrying fish codon-optimized *C. elegans fat-1* and *fat-2* were investigated on *S. aurata* intermediary metabolism and inflammation. Seventy-two hours post-treatment, a rapid enhancement of the expression levels of *C. elegans fat-1* (Δ15/*n*-3 desaturase) and *fat-2* (Δ12/*n*-6 desaturase) as well as the *n*-3 LC-PUFA content, particularly EPA and DHA, was observed in the liver of *S. aurata*. Studies examining the effects of *n*-3 LC-PUFAs on fish metabolism and immunity have primarily relied on dietary supplementation with varying levels of *n*-3 LC-PUFAs. However, this approach may underestimate differences in fatty acid oxidation, as *n*-3 LC-PUFAs are more prone to oxidation than unsaturated fatty acids. In contrast, the genetic approach used in this study prevented interferences due to fatty acid oxidation during diet manipulation and therefore avoided its putative negative effects on fish health and inflammation [15,16]. Moreover, unravelling the effects of *n*-3 LC-PUFAs on hepatic energy metabolism is essential for understanding and addressing growth retardation associated with decreased use of fish oil in aquafeeds [2].

It is well known that increased *n*-3 LC-PUFA values are associated with decreased plasma triglycerides and reduced cardiovascular risk in vertebrates [17]. Consistently, herein we showed that chitosan-mediated co-expression of *fat-1* and *fat-2* for 72 h in the fish liver caused an important reduction in blood triglycerides. Consistent with our results, *fat-1* transgenic models in mice [18], pigs [19], cattle [20], and zebrafish [10], also showed reduced circulating triglycerides and *n*-3 LC-PUFA accumulation. The triglyceride-lowering effect of *fat-1* and *fat-2* could be attributed to decreased production of very low-density lipoprotein (VLDL)-loaded triglycerides and enhanced triglyceride clearance, processes that may be facilitated by elevated *n*-3 LC-PUFA levels, as previously described in mice [17]. The *fat-1* transgenic models are also associated with reduced blood cholesterol [10,18,20]. Although not significant, a similar trend was found in fish expressing *fat-2* and *fat-1* + *fat-2*. Indeed, *n*-3 LC-PUFA was shown to decrease total cholesterol and low-density lipoprotein (LDL)-loaded cholesterol, while increasing high-density lipoprotein (HDL)-loaded cholesterol in mammals [21]. In the present study, glycemia was not significantly affected by any of the treatments assayed. However, the effect of *n*-3 LC-PUFA on blood glucose levels may depend on the fish species. Similarly to *S. aurata*, orange-spotted grouper (*Epinephelus coioides*) fed a soybean oil-based diet showed only marginal differences in blood glucose levels compared to fish fed fish oil-based diets [22]. In contrast, large yellow croaker (*Larimichthys crocea*) receiving 100% soybean oil as dietary lipid source showed higher blood glucose and lower hepatic glycogen levels than fish fed a fish oil diet [23].

Mutations in the *C. elegans fat-1* gene were shown to decrease 18:3*n*-3, 20:4*n*-3, and 20:5*n*-3 and increase 18:2*n*-6, 20:3*n*-6, and 20:4*n*-6 proportions [24], indicating its activity at Δ15 and Δ17 fatty acid substrates. Regarding *fat-2*, heterologous expression revealed its ability to sequentially insert double bonds at the Δ12 and Δ15 positions of fatty acids when a Δ9 double bond preexisted [25]. In line with these observations, herein the expression of *fat-1* increased the proportions of EPA, DHA, and total *n*-3 LC-PUFA, while *fat-2* also increased DHA and total *n*-3 LC-PUFA. Co-expression of *fat-1* and *fat-2* elevated EPA, DHA, total PUFA, and total *n*-3 and *n*-6 LC-PUFA, while a decreasing trend was found for the *n*-6 to *n*-3 ratio in *S. aurata* liver. Consistently, *fat-1* and *fat-2* transgenesis also enhances *n*-3 LC-PUFA synthesis in mammals and zebrafish [1,12,18,20,26].

To address the short-term effects of *C. elegans fat-1* and *fat-2* on transcription factors playing a key role in controlling the expression levels of rate-limiting enzymes in lipid and carbohydrate intermediary metabolism, the mRNA abundance of *srebf1*, *pparg*, *hnf4a*, *nr1h3*, and *chrebp* was investigated in the liver of *S. aurata* treated with chitosan-TPP-DNA nanoparticles. Similarly, as we reported after long-term sustained expression of *C. elegans fat-1* and *fat-2* in *S. aurata* [14] 72 h after a single nanoparticle administration to induce *fat-2* and *fat-1* + *fat-2* expression, a significant downregulation of hepatic *srebf1* was observed, which exerts a pivotal role in the transcriptional activation of lipogenic genes [27,28]. In agreement with our findings, transgenic models expressing *fat-1* and *fat-2* led to downregulation of *srebf1* in the liver [1,10,12]. Bearing in mind that DHA downregulates *srebf1* expression and promotes its protein degradation [29], increased levels of DHA in the liver of *S. aurata* expressing *C. elegans fat-1* and *fat-2* seem to be mainly responsible for downregulating *srebf1*. Concomitant to the alteration of liver fatty acid profile and decreased expression of *srebf1*, the hepatic expression of *fat-1*, *fat-2*, and *fat-1* + *fat-2* for 72 h downregulated genes related to *de novo* synthesis of fatty acids (*acaca*, *acacb, fasn*, and *scd1a*) and LC-PUFA (*fads2*) in *S. aurata*. Activation of Pparg has been shown to be involved in the regulation of lipogenesis in mice through the upregulation of genes such as *acaca*, *scd1*, and *fasn* [30]. Consistently, the short-term effects of *C. elegans fat-1* and *fat-2* on liver *srebf1* and lipogenic genes were reinforced by the downregulation of *pparg*. In support of this hypothesis, an increased dietary *n*-3/*n*-6 LC-PUFA ratio was also associated with reduced *pparg* levels in the liver of grass carp and large yellow croaker [31,32], while studies on rabbitfish (*Siganus canaliculatus*) hepatocytes showed that agonist-activation of Pparg upregulates the expression of *pparg*, *elovl5*, and *srebf1* [33]. Furthermore, *fat-1* transgenic zebrafish showed downregulation of hepatic *pparg* [10].

In transgenic animals, the expression levels of lipogenic genes were not only affected by *fat-1* and *fat-2* but also depended on the species and dietary lipid composition. For instance, *fat-1* transgenic zebrafish fed a low-fat diet presented higher hepatic expression of *fasn*, *acaca*, and *scd1* than fish fed with a high-fat diet [10]. Indeed, similar effects were reported in mice [34,35]. Although the differences were not statistically significant, we also observed lower mRNA levels of *elovl4a* and *elovl4b* in the liver of fish co-expressing *fat-1* and *fat-2*. However, we could not observe differences in *elovl5* expression, which may be due to the relatively short duration of the treatment. Previous reports in mice showed that EPA-enriched diets suppress the hepatic expression of *hmgcr*, a rate-limiting enzyme for cholesterol synthesis [36]. Accordingly, 72 h following nanoparticle administration, the expression of *fat-1* and *fat-2* downregulated *hmgcr* in *S. aurata* liver, while co-expression of *fat-1* + *fat-2* induced a non-significant trend toward decreasing blood cholesterol. Given that long-term sustained expression of *C. elegans fat-1* and *fat-2* significantly decreased both hepatic *hmgr* gene expression and blood cholesterol in *S. aurata* submitted to regular administration of chitosan-TPP-DNA nanoparticles [14], prolonged expression of *fat-1* and *fat-2* seems required to produce significant changes in the circulating levels of cholesterol.

As a carnivore, *S. aurata* exhibits low ability to metabolize dietary carbohydrates, which in turn leads to prolonged hyperglycemia both after a glucose load or when feeding high-carbohydrate diets [37]. However, we previously showed that long-term sustained expression of *C. elegans fat-2* (and *fat-1* + *fat-2*) improved glucose homeostasis in the liver of *S. aurata* through enhancing the activity of key enzymes in glycolysis and the PPP [14]. Consistently, 72 h following nanoparticle administration, the short-term effects of *fat-2* and *fat-1 + fat-2* expression in fish liver included the early upregulation of *pfkl* and *pklr* expression. In contrast, our findings suggest that a stronger stimulus (e.g., long-term sustained expression of *fat-2*) would be required to produce significant upregulation of *pfkfb1* expression and the subsequent increase in fructose-2,6-bisphosphate levels, leading to allosteric activation of *pfkl* and inhibition of *fbp1* [38]. This would ultimately enhance the glycolytic flux, as well as *g6pd* expression and PPP in the liver of *S. aurata*.

Hnf4a is a master regulator of liver metabolism through transcriptional regulation of target genes involved in glucose and lipid metabolism [39]. Consistent with previous reports indicating a major role of *hnf4a* and *nr1h3* in the long-term effects of sustained *fat-2* and *fat-1* + *fat-2* overexpression on key hepatic glycolytic enzymes in *S. aurata* [14], the present study showed that 72 h post-treatment with nanoparticles, *C. elegans fat-2* and *fat-1* + *fat-2*, also upregulated *hnf4a* expression at short-term in the liver of *S. aurata*. In agreement with our findings, dietary DHA supplement upregulated *hnf4* in mice liver [40]. Consistent with upregulation of *hnf4a* and enhancement of *pfkl* and *pklr* by *fat-2* and *fat-1* + *fat-2* in the present study, *hnf4a* expression decreased in *S. aurata* under conditions favoring gluconeogenesis versus glycolysis, such as treatment with streptozotocin and fasting [41]. Hnf4a regulates glucose and lipid metabolism mainly via *chrebp* and *nr1h3* [42]. While *nr1h3* expression showed a comparable but non-significant trend relative to *hnf4a*, co-expression of *fat-1* and *fat-2* also upregulated *chrebp* in the *S. aurata* liver. Hepatic activation of Chrebp is known to promote the glycolytic flux via increasing *pklr* expression, a mechanism that may contribute to *pklr* upregulation in the liver of *S. aurata* treated with *fat-1* and *fat-2* nanoparticles.

Based on the increased weight gain observed in *S. aurata* subjected to regular administration of chitosan-TPP-DNA nanoparticles to induce long-term sustained expression of *C. elegans fat-1* and *fat-2* [14], and considering that *fat-1* transgenesis prevents liver steatosis, glucose intolerance, and insulin resistance while exerting protective vascular effects through reduced inflammation [10,18,19,20,43], we hypothesized that elevated *n*-3 LC-PUFA levels and a decreased *n*-6/*n*-3 fatty acid ratio may contribute to improved fish health and enhanced immune status. Indeed, *n*-3 LC-PUFA play significant roles in the resolution of inflammation via downstream lipid mediators that can bind specific receptors and modulate the immune responses [44]. Tnfa, a cytokine secreted by macrophages, plays a pivotal role in diverse functions, including inflammatory responses, cell death, survival, proliferation, differentiation, and migration [45]. In the present study, a noteworthy reduction in *tnfa* mRNA levels was observed in *S. aurata* 72 h after the administration of *fat-2* and *fat-1* + *fat-2* nanoparticles, suggesting a potential attenuation of inflammatory reactions and an overall improvement of health condition. Consistently, in transgenic models expressing *C. elegans fat-1*, a similar trend was reported for the mRNA abundance and serum protein levels of this cytokine [46,47]. Changes in the proportions of certain fatty acids may result in a differential expression of *tnfa*. In this regard, resolvins derived from *n*-3 LC-PUFAs have been shown to resolve inflammation by inhibiting proinflammatory cytokines like *tnfa* during the inflammatory response [48]. Conversely, saturated fatty acids, such as stearic acid, correlate with increased expression of proinflammatory genes [49].

*S. aurata* treated with *fat-1*, *fat-2*, and *fat-1* + *fat-2* nanoparticles also presented a reduced hepatic expression of *nfkb1* (and a similar trend was found for *nfkb2*). Nfkb is a transcription factor involved in the transcriptional regulation of various genes related to the inflammatory response, including *il1*, *il6*, and *tnfa* [50]. Therefore, downregulation of *nfkb1* may possibly explain the reduced expression levels of *il6* and *tnfa* in the liver of fish co-expressing *fat-1* and *fat-2* for 72 h. In agreement with our findings, decreased nucleus phosphorylation and activity of Nfkb was observed in hepatic T cells of *fat-1* transgenic mice [47]. Furthermore, DHA and EPA were shown to decrease the expression levels and phosphorylation of Nfkb in the mammalian liver [51,52,53], while saturated fatty acids caused the opposite effects [54]. Conceivably, a lower inflammation status in fish expressing *fat-1* and *fat-2* may benefit growth performance. Hence, a recent study in grass carp (*Ctenopharyngodon idellus*) confirmed cytokine-mediated inhibition of growth hormone-induced actions [55], which is consistent with the elevated expression of these cytokines in fish fed diets unfavorable for growth [56,57]. In addition to the negative regulation of *n*-3 LC-PUFA on *nfkb* expression, we cannot rule out that *fat-1*- and *fat-2*-dependent downregulation of *nfkb1* in *S. aurata* may also result from molecular crosstalk with other affected transcription factors, notably the decreased expression of *srebf1* and the upregulation of *hnf4a*. In this regard, studies in cow hepatocytes have shown that reactive oxygen species produced as a result of *srebp1c* overexpression activate the Nfkb inflammatory pathway [58], while disruption of the Nfkb complex has been shown to decrease *srebp1c* protein levels in the mouse liver [59]. Furthermore, Hnf4a expression suppressed both basal and Tnfa-stimulated Nfkb activity in mammalian hepatic cells [60]. Indeed, Tnfa-mediated Nfkb activation was shown to interfere with the transactivation activity of Hnf4a in HepG2 cells [61].

## 4. Materials and Methods

### 4.1. Chitosan-TPP-DNA Nanoparticles

Fish codon-optimized *C. elegans fat-1* and *fat-2* DNA sequences were synthesized and ligated into the pSG5 plasmid (Agilent Technologies, Santa Clara, CA, USA). Chitosan-TPP nanoparticles complexed with empty pSG5 (used as a control), pSG5-FAT-1, or pSG5-FAT-2 were prepared by ionic gelation, as previously described [14]. Low-molecular-weight chitosan (Sigma-Aldrich, St. Louis, MO, USA) was dissolved at room temperature in acetate buffer solution (2 mg/mL) under magnetic stirring and filtered. After mixing 1 mL of 1 mg/mL pSG5, pSG5-FAT-1, or pSG5-FAT-2 with 4 mL of 0.84 mg/mL TPP (Sigma-Aldrich, St. Louis, MO, USA), the TPP-DNA solutions were added dropwise to 10 mL of the chitosan-acetate buffer solution to form chitosan-TPP-DNA nanoparticles. Nanoparticles were obtained by centrifugation at 36,000× *g* at 15 °C for 20 min and resuspended in 2 mL of 2% *w*/*v* mannitol, which was used as a cryoprotectant during subsequent lyophilization. After a freeze-drying cycle at −47 °C for 48 h, chitosan-TPP-DNA nanoparticles were stored at −20 °C until use. The particle size and *Z* potential were analyzed by dynamic light scattering and laser Doppler electrophoresis, respectively, employing a Zetasizer Nano ZS instrument (Malvern Instruments, Malvern, UK) with a 633 nm laser source. The nanoparticles were resuspended in 0.9% NaCl before being administered to *S. aurata*.

### 4.2. Animals

*S. aurata* juveniles with an average weight of 7.5 g (Avramar, Burriana, Spain) were transported to the aquatic animal facility at CCiTUB, where they were maintained at 23.0 °C in marine water 250 L tanks. Fish were maintained in a closed-circuit water system equipped with pump filters (eXperience 250, EHEIM, Deizisau, Germany), UV sterilizers (reeflexUV 800, EHEIM, Deizisau, Germany), and air pumps (air100, EHEIM, Deizisau, Germany). The photoperiod was 12 h light/dark. During acclimatization, a commercial diet (Microbaq 165, Dibaq, Segovia, Spain) was provided at a daily ratio of 5% body weight (BW), supplied at 9:00 and 17:00. For 2 weeks before beginning the experimental procedures and until the end of the process, the daily ration was kept at 3% of BW. Every 14 days, fish were weighed to readjust the feeding amount. Before handling and sampling, fish were anesthetized with 70 mg/L tricaine methansulfonate (MS-222, Thermo Fisher Scientific, Waltham, MA, USA) in marine water. Chitosan-TPP-DNA nanoparticles were injected intraperitoneally at a concentration of 10 μg DNA/g of BW and according to the following treatments: (1) chitosan-TPP-pSG5 (control with empty plasmid); (2) chitosan-TPP-pSG5-FAT-1; (3) chitosan-TPP-pSG5-FAT-2; (4) chitosan-TPP-pSG5-FAT-1 + chitosan-TPP-pSG5-FAT-2. Seventy-two hours later, fish were anesthetized and sacrificed by cervical section. The blood was collected and the liver was dissected out, immediately frozen in liquid nitrogen and kept at −80 °C until use. Ethical treatment of fish followed the guidelines of the Animal Welfare Committee of the University of Barcelona (proceeding #10811, Generalitat de Catalunya), in compliance with local (RD 53/2013) and EU (2010/63/EU) regulations.

### 4.3. Serum Metabolites

Serum glucose (Linear Chemicals, Montgat, Spain), triglycerides (Linear Chemicals, Montgat, Spain), and cholesterol (Spinreact, Sant Esteve de Bas, Spain) were measured according to the protocols of the corresponding commercial kit. Spectrophotometric determinations were performed in a Varioskan LUX multimode microplate reader (Thermo Fisher Scientific, Waltham, MA, USA).

### 4.4. Reverse Transcriptase-Coupled Quantitative Real-Time PCR (RT-qPCR)

Liver RNA from *S. aurata* was isolated with HigherPurity™ Tissue Total RNA Purification Kit (Canvax, Valladolid, Spain), and reverse-transcribed with Moloney murine leukaemia virus reverse transcriptase (Canvax, Valladolid, Spain) following manufacturer instructions. The mRNA levels of the genes listed in Table 2 were assayed with the QuantStudio™ 3 Real-Time PCR System (Thermo Fisher Scientific, Waltham, MA, USA) in 10 μL-reaction mixtures containing 0.4 μM of each primer (Table 2), 5 μL of SYBR Green (Thermo Fisher Scientific, Waltham, MA, USA), and 0.8 μL of diluted cDNA and sterilized milli-Q water. The amplification cycle was set at 95 °C for 10 min, followed by 40 cycles at 95 °C for 15 s and 62 °C for 1 min. Dissociation curves were performed to confirm single product amplification, while standard curves for determining the efficiency of the amplification reaction for each gene were generated by serial dilution of control cDNA. The geometric mean of *S. aurata* ribosomal subunit 18s (*18s*), β-actin (*actb*), and eukaryotic translation elongation factor 1 alpha (*eef1a*) was used to normalize gene expression levels. The standard ΔΔCT method was used to calculate variations in gene expression [62].

### 4.5. Fatty Acid Methyl Ester (FAME) Analysis

In total, 50 mg of liver and skeletal muscle samples were vortexed for 2 min in the presence of 1.5 mL of ice-cold methanol:chloroform solution 2:1 (*v*/*v*) in glass tubes. This step was followed by the addition of 0.5 mL of ice-cold chloroform and 0.5 mL of ice-cold milli-Q water, vortexing for 30 s after each addition. After centrifuging at 1000× *g* and 4 °C for 15 min, the middle layer was transferred to opaque vials and submitted to solvent volatilization at 25 °C under gentle nitrogen flux until only oil residue was present. The residue was dissolved by vortexing in 0.5 mL of *n*-hexane, followed by the addition of 0.2 mL of 2 M potassium hydroxide in methanol solution, vortexing for 30 sec, incubation at 25 °C for 3 min, and centrifugation for 5 min at 2000× *g* and 4 °C. The upper phase was transferred to gas chromatography vials and kept at −20 °C until gas chromatography was performed.

Fatty acid profiles in liver and skeletal muscle samples were analyzed by gas chromatography with flame ionization detection using GC-2025 (Shimadzu, Kyoto, Japan) with capillary column BPX70, 30 m × 0.25 mm × 0.25 mm (Trajan Scientific and Medical, Ringwood, Australia). The oven temperature was held for 1 min at 60 °C and then raised to 260 °C at a rate of 6 °C/min. Injector (AOC-20i, Shimadzu, Kyoto, Japan) and detector temperatures were set at 260 °C and 280 °C, respectively. One microliter of the sample was injected with helium as carrier gas and a split ratio of 1:20. Supelco 37 Component FAME Mix (Sigma-Aldrich, St. Louis, MO, USA) was included as a reference for fatty acid identification. Gas chromatography data were expressed as percentage of content, calculated with OpenchromV13 (Lablicate, Hamburg, Germany).

### 4.6. Statistics

A one-way ANOVA was employed to determine significant differences among fish treated with pSG5 (control), FAT-1, FAT-2, and FAT-1 + FAT-2. When the ANOVA was significant (*p* < 0.05), the Duncan post hoc test was applied to perform pairwise statistical comparisons between the experimental groups and identify homogeneous subsets of means (*p* < 0.05). Statistical analyses were conducted using SPSS software Version 26 (IBM, Armonk, NY, USA).

## 5. Conclusions

**Figure 8:**
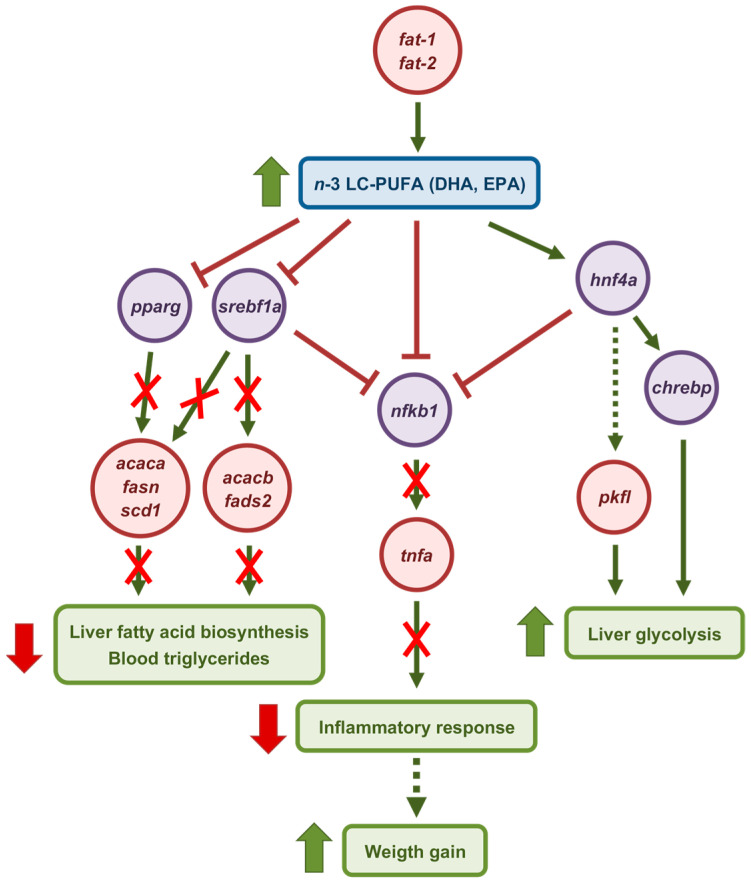
Schematic representation of the short-term molecular events triggered by co-expressing fish codon-optimized *C. elegans fat-1* and *fat-2* in the liver of *S. aurata*. Seventy-two h after the administration of chitosan-TPP-DNA nanoparticles, the expression of fish codon-optimized *C. elegans fat-1* and *fat-2* stimulated the hepatic synthesis of *n*-3 LC-PUFA, particularly EPA and DHA. This event upregulated transcription factors and key enzymes involved in glycolysis, while downregulating those associated with fatty acid biosynthesis and proinflammatory factors, thereby potentially improving growth performance. Red crossed arrows indicate loss of activating effect. Dotted arrows indicate potential interactions.

The present study discloses the short-term molecular events triggered by chitosan-TPP-DNA nanoparticles expressing fish codon-optimized *C. elegans fat-1* and *fat-2* in the liver of *S. aurata*. Co-expression of both enzymes resulted in the most pronounced effects across the majority of the parameters analyzed (Figure 8). In addition to elevating *n*-3 LC-PUFA such as EPA and DHA in the liver, 72 h post-treatment with *fat-1* and *fat-2* nanoparticles suppressed the hepatic expression of *srebf1*, *pparg*, and genes encoding key enzymes in *de novo* lipogenesis and LC-PUFA synthesis, while *fat-2* and *fat-1* + *fat-2* upregulated *hnf4a*, *chrebp*, and rate-limiting enzymes in glycolysis. Notably, our data show that *C. elegans fat-1* and *fat-2* exerted rapid effects, within less than 72 h, by enhancing EPA and DHA levels in the liver of *S. aurata*, which in turn may have determined downregulation of proinflammatory factors such as *tnfa* and *nfkb1*. Altogether, our findings suggest that in addition to enabling the production of cultured fish rich in *n*-3 LC-PUFAs for human consumption, *fat-1* and *fat-2* expression may impact the health status and growth of fish. Future studies will be required to assess the extent to which the observed changes could benefit the growth performance of farmed fish.

## Figures and Tables

**Table 1:** Effect of chitosan-TPP nanoparticles complexed with pSG5 (control), pSG5-FAT-1, pSG5-FAT-2, and pSG5-FAT-1 + pSG5-FAT-2 on the fatty acid composition of *S. aurata* liver.

Fatty Acid	pSG5	FAT-1	FAT-2	FAT-1 + FAT-2
14:0	5.7 ^a^ ± 0.9	7.2 ^ab^ ± 0.1	8.7 ^b^ ± 0.7	7.4 ^ab^ ± 0.7
16:0	19.0 ± 2.9	20.3 ± 0.9	22.3 ± 0.5	24.4 ± 1.4
18:0	3.0 ± 0.7	3.2 ± 0.6	2.6 ± 0.3	4.0 ± 0.4
16:1*n*-7	2.6 ^a^ ± 0.7	4.2 ^b^ ± 0.3	5.4 ^b^ ± 0.2	4.9 ^b^ ± 0.3
18:1*n*-9	14.4 ± 2.7	16.1 ± 1.8	17.6 ± 1.4	18.8 ± 0.7
18:2*n*-6	16.0 ^a^ ± 3.3	18.2 ^ab^ ± 1.2	20.2 ^ab^ ± 1.4	24.0 ^b^ ± 1.1
18:3*n*-3	0.8 ± 0.3	1.3 ± 0.4	1.1 ± 0.2	1.0 ± 0.2
20:5*n*-3	1.6 ^a^ ± 0.4	2.4 ^b^ ± 0.1	2.3 ^ab^ ± 0.1	2.5 ^b^ ± 0.2
22:6*n*-3	1.7 ^a^ ± 0.2	2.6 ^b^ ± 0.2	2.6 ^bc^ ± 0.2	3.2 ^c^ ± 0.2
SFA	28.0 ± 4.6	30.9 ± 1.4	33.7 ± 0.7	36.1 ± 1.8
MUFA	17.7 ± 2.7	21.1 ± 2.2	23.8 ± 1.3	23.8 ± 0.7
PUFA	20.6 ^a^ ± 3.7	25.2 ^ab^ ± 1.5	27.0 ^ab^ ± 1.9	31.4 ^b^ ± 1.3
*n*-3	4.1 ^a^ ± 0.6	6.2 ^b^ ± 0.4	6.0 ^b^ ± 0.5	6.9 ^b^ ± 0.3
*n*-6	16.5 ^a^ ± 3.2	18.8 ^ab^ ± 1.2	20.7 ^ab^ ± 1.4	24.3 ^b^ ± 1.1
*n*-9	14.8 ± 2.7	16.7 ± 1.9	18.3 ± 1.3	19.0 ± 0.6
*n*-6/*n*-3	4.0 ± 0.5	3.0 ± 0.2	3.5 ± 0.2	3.5 ± 0.1

Data are expressed as a percentage of total fatty acids and represented as the mean ± SEM (*n* = 4). Only fatty acids exceeding 1% are shown. Different letters indicate distinct homogeneous subsets determined by the post hoc test (*p* < 0.05).

**Table 2:** Primer sequences used for RT-qPCR in the present study.

Gene	Forward Sequences (5′ to 3′)	Reverse Sequences (5′ to 3′)	GenBank Accession
*acaca*	CCCAACTTCTTCTACCACAG	GAACTGGAACTCTACTACAC	JX073712
*acacb*	TGACATGAGTCCTGTGCTGG	GCCTCAGTTCGTATGATGGT	JX073714
*actb*	CTGGCATCACACCTTCTACAACGAG	GCGGGGGTGTTGAAGGTCTC	X89920
*chrebp*	CTTCGACACGGTGAACAGGA	AGGGACATGCAGTCGAACAG	NC044199
*eef1a*	CCCGCCTCTGTTGCCTTCG	CAGCAGTGTGGTTCCGTTAGC	AF184170
*elovl4a*	AAGAACAGAGAGCCCTTCCAG	TGCCACCCTGACTTCATTG	MK610320
*elovl4b*	TCTACACAGGCTGCCCATTC	CGAAGAGGATGATGAAGGTGAC	MK610321
*elovl5*	GGGATGGCTACTGCTCGACA	CAGGAGAGTGAGGCCCAGAT	AY660879
*fads2*	CACTATGCTGGAGAGGATGCC	TATTTCGGTCCTGGCTGGGC	AY055749
*fasn*	GTAGAGGACACGCCCATCGAT	TGCGTATGACCTCTTGGTGTGCT	JQ277708
*fat-1*	TTCAACCCCATTCCTTTCAGCG	TAGGCGCACACGCAGCAGCA	ON374024
*fat-2*	AAGAGGACTACAACAACAGAACCGCCA	CGAACAGTCTGCTCCAAGGCCAA	ON374025
*fbp1*	CAGATGGTGAGCCGTGTGAGAAGGATG	GCCGTACAGAGCGTAACCAGCTGCC	AF427867
*g6pd*	TGATGATCCAACAGTTCCTA	GCTCGTTCCTGACACACTGA	JX073711
*hmgcr*	ACTGATGGCTGCTCTGGCTG	GGGACTGAGGGATGACGCAC	MN047456
*hnf4a*	GTGGACAAAGACAAGCGAAATC	GCATTGATGGATGGTAAACTGC	FJ360721
*il1b*	CGTCATCGCCATGGAGAGGT	TGAGCTGGTTTTGCAGTGCG	AJ277166
*il6*	CGGAGCAGCATCGTCACTTT	TTCTGCATGGCACACACAAT	EU244588
*myd88*	GCGACGCCTGTGACTTTCAG	GGGTGTAGTCGCAGAGGGTG	XM_030399037
*nfkb1*	GCTGCTGCTCGGGATCAAAC	GTCCACACTGAGCCACTGGA	XM_030396749
*nfkb2*	GTGTGTTGCTGCCGTGTGAC	GTCTGTCCGTCTGCTCCCTC	XM_030441891
*nr1h3*	GCATCTGGACGAGGCTGAATAC	ACTTAGTGTGCGAAGGCTCACC	FJ502320
*pck1*	CAGCGATGGAGGAGTGTGGTGGGA	GCCCATCCCAATTCCCGCTTCTGTGCTCCGGCTGGTCAGTGT	AF427868
*pfkfb1*	TGCTGATGGTGGGACTGCCG	CTCGGCGTTGTCGGCTCTGAAG	U84724
*pfk1*	TGCTGGGGACAAAACGAACTCTTCC	AAACCCTCCGACTACAAGCAGAGCT	KF857580
*pklr*	CAAAGTGGAAAGCCGGCAAGGG	GTCGCCCCTGGCAACCATAAC	KF857579
*pparg*	TGCGAGGGCTGTAAGGGTTTC	GTTTCTCCTTCTCCGCCTGGG	AY590304
*srebf1*	CAGCAGCCCGAACACCTACA	TTGTGGTCAGCCCTTGGAGTTG	JQ277709
*scd1a*	TCCCTTCCGCATCTCCTTTG	TTGTGGTGAACCCTGTGGTCTC	JQ277703
*tnfa*	GTCTTCCGCCCCTCAGATCC	GAAAGCCGAAGCATGAGCCC	AJ413189
*18s*	TTACGCCCATGTTGTCCTGAG	AGGATTCTGCATGATGGTCACC	AM490061

## Data Availability

Data will be made available from the corresponding author upon reasonable request.

## Abbreviations

The following abbreviations are used in this manuscript:

ALA	α-Linolenic acid
DHA	Docosahexaenoic acid
EPA	Eicosapentaenoic acid
LA	Linoleic acid
OA	Oleic acid
BW	Body weight
TPP	Tripolyphosphate
n-3 LC-PUFA	omega-3 long-chain polyunsaturated fatty acids
